# Effect of Experimental Parameters on Cavitation Dose in Ultrasonic Baths via Modified Aluminum Foil Test

**DOI:** 10.3390/molecules31081291

**Published:** 2026-04-15

**Authors:** Svetlana Saikova, Diana Nemkova, Anton Krolikov

**Affiliations:** 1 School of Non-Ferrous Metals, Siberian Federal University, Svobodny, 79, 660041 Krasnoyarsk, Russia; dsaykova@sfu-kras.ru; 2 Institute of Chemistry and Chemical Technology, Federal Research Center “Krasnoyarsk Science Center of the Siberian Branch of the Russian Academy of Sciences”, Akademgorodok, 50/24, 660036 Krasnoyarsk, Russia; antonkrolikov@mail.ru

**Keywords:** sonication, dispersion, aluminum foil, cavitation erosion, solvent properties

## Abstract

Ultrasonic cavitation is a key mechanism in the dispersion and erosion of solid materials in liquids; however, the influence of processing conditions and medium properties on its efficiency in ultrasonic baths remains poorly systematized. Despite the widespread use of ultrasonic baths in materials processing, general optimization principles are lacking, and operating parameters are typically determined empirically for each system. In this work, cavitation activity was quantitatively assessed using an aluminum foil erosion test, with the foil clamped in a plastic frame to evaluate the mechanical effects of cavitation. The effects of ultrasonic power, frequency, treatment time, temperature, solvent nature, and vessel material on the foil mass loss were systematically investigated. The results demonstrate that both the instrumental parameters and physicochemical properties of the dispersion medium, including viscosity and surface tension, significantly affect the cavitation activity. Solvents with lower cavitation thresholds and favorable acoustic properties promote more intense erosion, while the vessel material and geometry also influence energy transmission to the liquid. This study provides a systematic framework for assessing the cavitation dose in ultrasonic baths and offers practical guidelines for optimizing ultrasonic dispersion processes and improving their reproducibility.

## 1. Introduction

Ultrasonic irradiation of liquids generates a variety of physical and chemical effects, including heating, mixing and the formation of transient microscopic hotspots that can drive high-energy reactions [1,2,3]. Two principal modes of ultrasound delivery are commonly employed: direct (via a sonotrode or ultrasonic probe) [4,5,6,7,8,9,10,11,12] and indirect (via an ultrasonic bath) [13,14,15,16,17].

Direct sonication typically relies on a Langevin transducer immersed in the liquid [12]. Acoustic energy is concentrated in the immediate vicinity of the sonotrode tip, enabling efficient dispersion of bulk materials. Several comparative studies have demonstrated the superiority of the probe over the bath for applications such as Cu powder dispersion [16] and biofilm removal [17]. However, prolonged or high-power operation of immersion probes causes significant heating of the medium and erosion of the titanium or aluminum tip, leading to contamination of the product with metallic debris [18].

Indirect sonication, using ultrasonic baths, generates a diffuse, relatively low-intensity cavitation field that is distributed throughout the liquid volume. Although the attainable pressure amplitudes are lower than those achieved near a probe horn, the absence of direct contact between the transducer and the processed medium eliminates both excessive local heating and erosion-related contamination. As a result, ultrasonic baths are particularly attractive for the preparation of high-purity nanomaterials and for applications requiring reproducible treatment of multiple samples.

Ultrasound can promote the formation of nanostructures through two distinct mechanisms: chemical (sonochemical) and physical (mechanical). Sonochemical effects arise from the extreme conditions generated inside collapsing cavitation bubbles, where temperatures of several thousand Kelvin and pressures of hundreds of bar are reached [19]. These conditions induce bond cleavage and enable the synthesis of a wide range of nanostructured materials, including metal oxides such as TiO_2_, ZnO and Fe_3_O_4_ [6,7,8,13].

In contrast, physical effects originate from shock waves and high-velocity microjets produced during asymmetric bubble collapse. These mechanical impacts can fragment brittle solids into smaller particles and are widely exploited for the exfoliation and dispersion of layered materials, metals and ceramics [4,5,9,10,14]. For example, probe sonication at 24 kHz has been used to disintegrate Al_3_Ti, Al_3_V, Si and Al_3_Zr crystals [4,5]; gold foil and powder were converted into spherical nanoparticles with sizes of approximately 12–14 nm after prolonged probe sonication [9]; and nanosheets of NiMnO_3_, MoS_2_ and Cr_2_S_3_ were obtained from bulk powders using either ultrasonic probes [10,19,20] or ultrasonic baths [14].

The efficiency of ultrasonic dispersion is governed by the intensity of the shock waves emitted during bubble collapse, which depends on the bubble radius at the moment of implosion [21]. Bubble dynamics are controlled by both the operating parameters of the ultrasonic device, such as frequency and acoustic power [22,23], and the physicochemical properties of the liquid medium, including viscosity, surface tension, vapour pressure and gas solubility [24,25,26,27]. Liquids with low viscosity and low surface tension facilitate cavitation at lower energy thresholds; consequently, solvent choice represents a critical, yet often under-reported, experimental variable.

Numerous experimental approaches have been developed to characterize cavitation activity. These include measurements of primary effects, such as pressure and temperature during bubble collapse [4,28,29], as well as secondary effects, including chemical dosimetry based on KI oxidation [25,30,31,32], sonoluminescence [33,34,35,36,37,38] and cavitation-assisted dispersion of solid materials [39,40,41,42,43,44]. KI dosimetry is widely employed owing to its simplicity and good reproducibility; however, it primarily reflects the chemical action of cavitation rather than its mechanical impact. In contrast, the aluminum foil erosion test can provide a direct and quantitative measure of the mechanical action of cavitation, which is the key mechanism responsible for the fragmentation and dispersion of solid particles. This method is simple, inexpensive and enables straightforward visualization of the spatial distribution of cavitation intensity. Under cavitation conditions, thin aluminum foil is perforated within minutes, and the resulting erosion patterns directly map the locations of cavitation foci [44]. Several studies have employed aluminum foil in ultrasonic baths to qualitatively characterize cavitation activity through erosion pattern analysis [40,44,45,46,47]. In the present work, we extend this approach by introducing foil mass loss as a quantitative measure of the cavitation dose.

Despite the widespread use of ultrasonic baths in materials processing, systematic investigations into how combined experimental parameters—such as ultrasonic frequency, electrical power, solvent properties, vessel material and temperature—govern the cavitation dose delivered to a sample are still lacking. Moreover, studies on solvent-assisted dispersion typically rely on arbitrary experimental conditions, and systematic comparisons of solvents under identical acoustic and geometrical parameters are rarely performed. This lack of standardization contributes to the well-recognized reproducibility challenges in sonochemistry [39]. To the best of our knowledge, few studies have systematically examined quantitative correlations between cavitation dose in ultrasonic baths and solvent properties under identical acoustic and geometrical conditions.

Here, we address this gap by employing a modified aluminum foil erosion test to quantitatively assess cavitation activity under controlled variation of key experimental parameters. A custom-designed foil holder was developed to position aluminum foil samples perpendicular to the transducer surface, enabling consistent exposure across pressure nodes and antinodes. Foil mass loss was adopted as a robust quantitative metric of cavitation dose. Using two commercial ultrasonic baths operating at 35 and 68 kHz (nominal power density 60 W L^−1^), we systematically mapped the cavitation field and investigated the influence of ultrasonic frequency, electrical power, exposure time, liquid temperature, solvent type (water, methanol, ethanol, isopropanol and dimethyl sulfoxide (DMSO)) and vessel material. The resulting dataset establishes clear relationships between solvent properties—vapour pressure, surface tension, viscosity and gas solubility—and the measured cavitation dose, and provides practical guidelines for the rational selection of operating conditions in ultrasonic bath-assisted dispersion processes.

## 2. Results and Discussion

### 2.1. Mapping of Cavitation Activity in Ultrasonic Baths

The spatial inhomogeneity of the ultrasonic field in cleaning baths arises from interference between waves reflected from the tank walls and the liquid surface, combined with flexural vibration modes of the tank. These phenomena create a standing wave pattern with distinct regions of high and low acoustic pressure, corresponding to zones of strong and weak cavitation activity [15,37].

**Figure 1:**
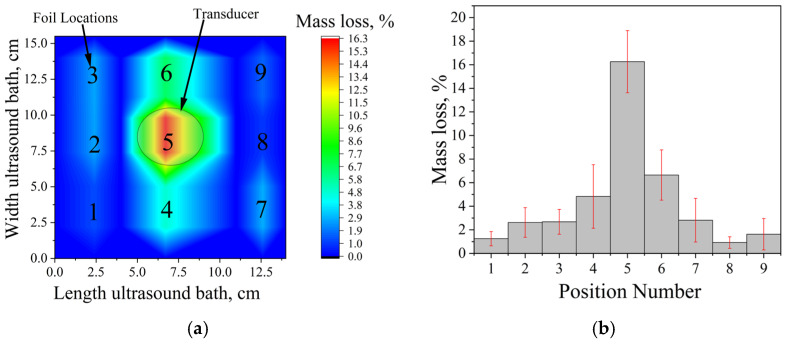

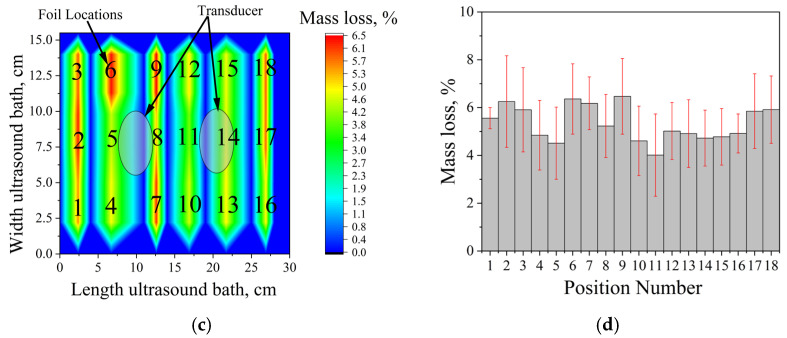
Cavitation maps of ultrasonic baths obtained by aluminum foil erosion after 5 min at 25 °C and 60 W·L^−1^: (**a**) spatial distribution of mass loss in the Ultrasonic cleaner (35 kHz); (**b**) corresponding mass loss profile as a function of position; (**c**) spatial distribution in the Vilitek VBS-13DS bath (68 kHz); (**d**) corresponding mass loss profile, data are presented as mean ± standard deviation (SD), *n* = 7 independent experiments. Error bars represent the SD.

To visualize this distribution and identify the optimal sample position for subsequent experiments, cavitation mapping was performed using the aluminum foil erosion test. Pre-weighed foil sheets (12 × 2.5 cm, thickness 10 μm) were clamped in a wooden frame and positioned vertically at different locations within each bath. After 5 min of sonication at 25 °C and a nominal power density of 60 W∙L^−1^, the foils were removed, rinsed, dried, and weighed. The mass loss at each position was used as a quantitative measure of local cavitation activity, and the obtained values were interpolated to construct two-dimensional cavitation maps (Figure 1), identifying “hot spots” of strong cavitation and regions of weak or absent activity. Such mapping is essential for optimizing experimental conditions, ensuring reproducibility, and avoiding localized overheating or underexposure.

For the Ultrasonic cleaner (35 kHz; single 45 mm transducer), cavitation activity is strongly concentrated directly above the transducer, with mass loss decreasing sharply with horizontal distance from this hot spot (Figure 1a,b). Accordingly, in all subsequent experiments with this bath, the sample vessel was placed centrally over the transducer.

The Vilitek VBS-13DS bath (68 kHz; two 45 mm transducers) exhibits a considerably more uniform cavitation field, with no pronounced peaks or deep minima across the tested positions (Figure 1c,d). Based on this mapping, the sample was positioned in the geometric center of the bath, equidistant from both transducers. This fixed location, corresponding to the region of highest mechanical cavitation impact, was used throughout all further experiments to ensure reproducible exposure conditions and minimize position-induced variability.

### 2.2. Effect of the Vessel Material on the Cavitation Dose

The influence of the vessel material on the cavitation dose was investigated using the Ultrasonic cleaner bath (35 kHz; 60 W∙L^−1^) at 25 °C for 5 min. Three cylindrical vessels (150 mL) were tested: two glass vessels (1, 2) of different glass types and wall thickness and one polypropylene vessel (3), all filled with 100 mL of distilled water. Their relevant physical properties are summarized in Table 1.

**Figure 2:**
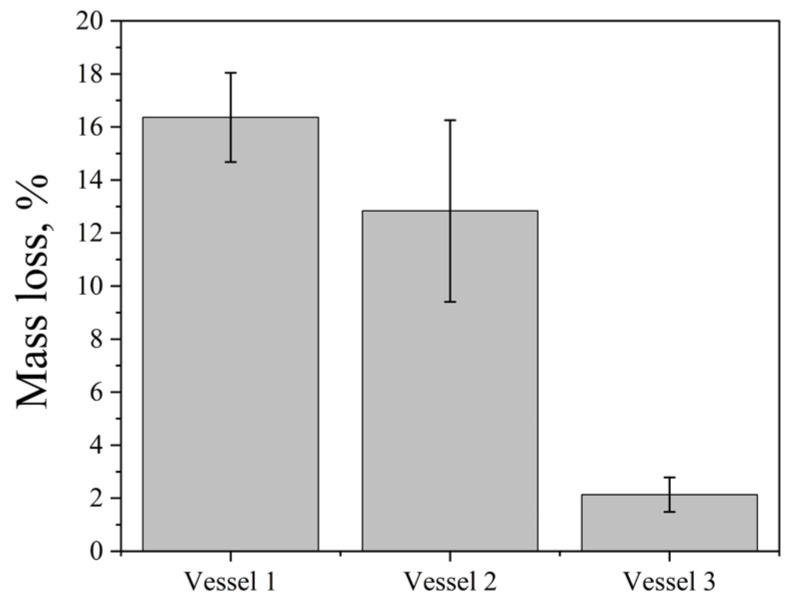
Mass loss of aluminum foil after ultrasonic treatment in vessels of different materials (35 kHz, 60 W·L^−1^, 25 °C, 5 min). Data are presented as mean ± standard deviation (SD), *n* = 12 independent experiments. Error bars represent the SD.

The mass loss of aluminum foil was greater in glass vessels than in the polypropylene vessel (Figure 2). Glass, which has a lower attenuation coefficient (typically 0.01–0.1 dB/cm, depending on ultrasound frequency and glass composition) and higher stiffness (elastic modulus 48–83 GPa), transmits ultrasonic energy through the vessel walls more efficiently than polypropylene. The higher stiffness minimizes energy dissipation through wall deformation, while the lower attenuation reduces absorption of acoustic waves within the material, resulting in more effective delivery of cavitation energy to the liquid inside.

Thicker walls (vessel 2) increase the acoustic path length, leading to greater energy loss through scattering and absorption within the wall material and consequently reducing sound intensity. It results in lower aluminum foil erosion in vessel 2 compared to vessel 1. Therefore, all subsequent experiments were performed using vessel 1.

### 2.3. Effect of Ultrasound Frequency and Power on the Cavitation Dose

Table 2 presents the effect of ultrasound frequency on aluminum foil mass loss. The experiments were carried out at 25 °C with a nominal power density of 60 W·L^−1^ for 5 min. Different commercial ultrasonic baths were used for these experiments (Table 2); however, both employ piezoelectric transducers made of lead zirconate titanate (PZT)—the standard material for such equipment. While the geometric dimensions of the baths differed, the applied specific power (power per unit volume) was the same, and all other experimental conditions were identical. Thus, the primary systematic difference between them was the operating frequency (35 kHz vs. 68 kHz), allowing a qualitative comparison of the influence of frequency on the cavitation dose. Increasing the ultrasound frequency from 35 to 68 kHz resulted in an approximately 60% decrease in mass loss. The observed decrease at higher frequency is consistent with the general tendency reported in the literature and is commonly attributed to a substantial reduction in the average cavitation bubble radius with increasing ultrasound frequency [23,36,50]. A smaller maximum bubble radius results in a lower amount of energy accumulated during the expansion stage and, consequently, in a reduced intensity of shock waves generated upon bubble collapse [21].

**Figure 3:**
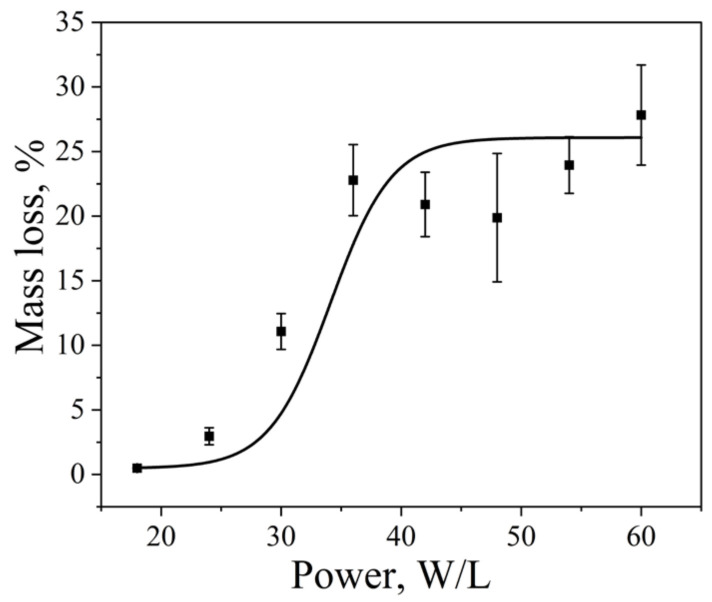
Aluminum foil mass loss as a function of ultrasonic power density in a Vilitek VBS-4DP ultrasonic bath (68 kHz, 25 °C, 15 min). Data are presented as mean ± standard deviation (SD), *n* = 12 independent experiments. Error bars represent the SD.

The dependence of aluminum foil mass loss on ultrasonic power was investigated using a Vilitek VBS-4DP ultrasonic bath (68 kHz) at 25 °C for an exposure time of 15 min. With increasing ultrasonic power, the dispersion efficiency increased. A longer sonication time was chosen in this case to ensure measurable foil mass loss over the entire range of applied power densities. The cavitation dose, expressed as foil mass loss, increased from 2.9 ± 0.7% at 20 W·L^−1^ to 20.9 ± 2.5% at 35 W·L^−1^ (Figure 3). At power densities above 35 W·L^−1^, the mass loss reached a plateau, while the standard deviation increased, indicating enhanced temporal and spatial variability of cavitation activity under these conditions. This behavior can be rationalized in terms of changes in bubble population and size with increasing acoustic power. For instance, at 200 kHz, an increase in power from 63.45 to 115.8 W·L^−1^ was reported to raise the bubble number density from 3.8 × 10^11^ to 4.92 × 10^11^ m^−3^ [23]. Similarly, at 1056 kHz, increasing the power from 2 to 10 W resulted in an increase in the average bubble radius from 1.8 to 4.5 μm [36]. Although these studies were conducted at higher frequencies, they illustrate general trends in the evolution of bubble population and size with increasing acoustic intensity. Further increases in power density did not lead to a significant additional increase in foil mass loss. At high acoustic intensities, the formation of dense cavitation clouds and bubble coalescence can occur, leading to acoustic shielding of the bulk liquid and limiting the effective transmission of ultrasound throughout the volume, thereby hindering further development of cavitation [31,51].

### 2.4. Effect of Dissolved Gas Concentration on the Cavitation Dose

To investigate the influence of dissolved gas concentration on cavitation activity, three liquid media were prepared: distilled water (control), degassed distilled water, and CO_2_-saturated water. Degassed water was obtained by boiling distilled water for 30 min, followed by cooling to room temperature under ambient conditions. CO_2_-saturated water was prepared by bubbling carbon dioxide through distilled water at a pressure of 8 atm and a temperature of 15 °C. The total dissolved gas concentrations for degassed (1) and distilled (2) water were obtained from the literature [52,53]. For CO_2_-saturated water (3), the gas concentration was determined experimentally using a gas analyzer (“OKA 92MT”) immediately after saturation. Distilled water was saturated with carbon dioxide at a pressure of 8 atm and a temperature of 15 °C. All experiments were carried out in an Ultrasonic cleaner bath operating at 35 kHz with a nominal power density of 60 W·L^−1^ at 25 °C for 5 min.

The results are summarized in Table 3. Degassing of the liquid led to a noticeable decrease in the dispersion efficiency, as reflected by the reduced aluminum foil mass loss. This effect is attributed to the depletion of pre-existing gas nuclei in the liquid, which serve as cavitation nucleation sites. Their absence suppresses bubble formation, resulting in lower cavitation activity and diminished mechanical erosion of the foil [54,55].

In contrast, a high concentration of dissolved gas also caused a pronounced decrease in foil mass loss. Under these conditions, the cavitation field is characterized by the formation of a large number of bubbles with a high overall void fraction. Such bubble populations efficiently scatter and absorb the incident ultrasonic waves, leading to acoustic shielding of the bulk liquid and a reduction in the effective cavitation intensity available for erosion [56]. As a result, the dependence of aluminum foil mass loss on dissolved gas concentration exhibits a non-monotonic character, with a maximum at moderate gas concentrations.

### 2.5. Effect of Ultrasonic Treatment Time on the Cavitation Dose

**Figure 4:**
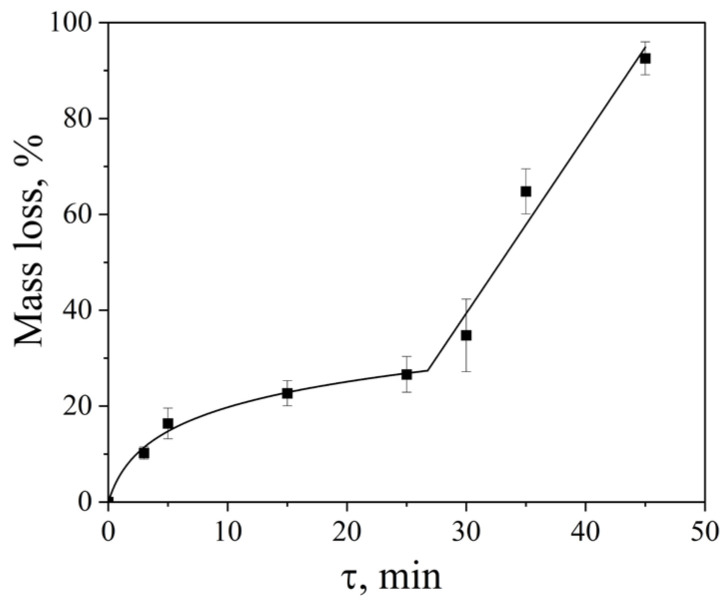
Aluminum foil mass loss as a function of ultrasonic treatment time (Ultrasonic cleaner, 35 kHz, 60 W·L^−1^, 25 °C). Two distinct regimes are observed: logarithmic growth (3–30 min) and linear behavior (>30 min). Data are presented as mean ± standard deviation (SD), *n* = 12 independent experiments. Error bars represent the SD.

The influence of ultrasonic treatment time on the cavitation dose was investigated using an Ultrasonic cleaner bath operating at 35 kHz with a nominal power density of 60 W·L^−1^ at 25 °C. With increasing sonication duration, the dispersion efficiency increased progressively. The dependence of aluminum foil mass loss on treatment time exhibited a complex, two-stage character (Figure 4).

In the initial stage (3–30 min), the foil mass loss increased in a logarithmic manner, from 10 ± 1% to 34 ± 7%. In this regime, the dominant mechanism of material degradation is associated with fatigue-related processes resulting from repeated mechanical loading of the solid surface by shock waves and microjets generated during cavitation bubble collapse [4,5]. Such cyclic stresses promote the gradual growth of pre-existing cracks and structural defects until a critical size is reached, ultimately leading to brittle fracture of the foil.

**Figure 5:**
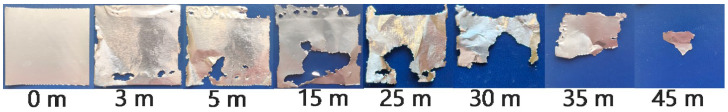
Photographs of aluminum foil samples after ultrasonic treatment for 3 to 45 min, illustrating progressive erosion and eventual disintegration of the foil.

After approximately 30 min of ultrasonic treatment, the erosion behavior transitioned to a linear regime. At this stage, progressive disintegration at the foil edges caused the sample to detach from the holder and disperse into the bulk liquid as freely suspended fragments (Figure 5). This change in the physical state of the sample—from a fixed foil to mobile fragments—modifies the erosion mechanism and accounts for the observed linear dependence of mass loss on treatment time.

It should also be noted that prolonged sonication at extended treatment times (typically beyond 15–20 min) led to increased variability in the experimental results. This behavior is attributed to partial overheating of the liquid and fluctuations in transducer performance, which can destabilize the cavitation field and affect the reproducibility of cavitation-induced erosion during long-term operation of ultrasonic baths.

### 2.6. Effect of Solvent Temperature on the Cavitation Dose

**Figure 6:**
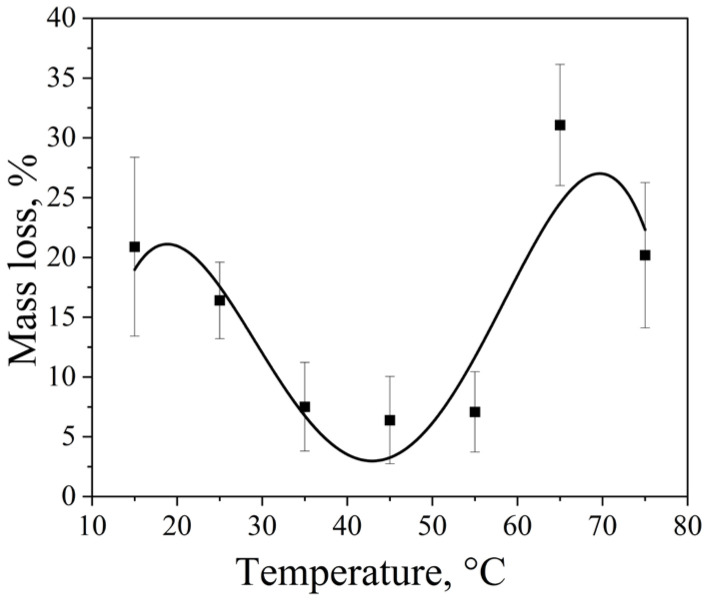
Aluminum foil mass loss as a function of water temperature (Ultrasonic cleaner, 35 kHz, 60 W·L^−1^, 5 min). Data are presented as mean ± standard deviation (SD), *n* = 12 independent experiments. Error bars represent the SD.

The influence of water temperature on cavitation erosion efficiency was investigated using an ultrasonic cleaner operating at 35 kHz with a nominal power density of 60 W·L^−1^ and a treatment time of 5 min, while keeping all other parameters constant (for comparison, a similar experiment was conducted under comparable conditions using a Vilitek VBS 13DS ultrasonic bath (68 kHz, 60 W·L^−1^, 5 min) (Figure S1). As shown in Figure 6 and Figure S1, irrespective of the equipment used, the mass loss of the aluminium foil exhibits a non-monotonic, biphasic dependence on temperature, with two distinct maxima at 15 °C and 65 °C and a pronounced minimum in the intermediate range (35–55 °C). This complex behaviour arises from the coupled temperature dependence of several key physicochemical properties of water, including surface tension, viscosity, saturated vapour pressure, and the concentration of dissolved gases (Table 4).

At low temperatures (15 °C), water is characterised by relatively high surface tension and viscosity, low saturated vapour pressure, and a high concentration of dissolved gases (in accordance with Henry’s law). These conditions favour the formation and stability of gas-containing cavitation nuclei. During acoustic excitation, such nuclei give rise predominantly to gas-filled cavitation bubbles. Because the vapour pressure is low, vapour content within the bubbles remains limited, and their collapse is governed mainly by gas compression. As a result, damping effects associated with vapour condensation are minimal, leading to highly energetic bubble collapse, strong shock wave emission, and enhanced mechanical impact on the solid surface, which manifests as high erosion efficiency.

At elevated temperatures (65 °C), both surface tension and viscosity decrease, which lowers the cavitation threshold and promotes the formation of a larger number of cavitation bubbles. However, the saturated vapour pressure increases sharply, and the solubility of gases decreases, resulting in a shift towards vapour-dominated cavitation. Under these conditions, cavitation bubbles contain a significant fraction of vapour, which undergoes condensation during collapse and effectively dampens the compression process. This reduces the peak pressures and temperatures achieved during bubble collapse and weakens the associated shock waves. The observed erosion maximum at 65 °C can therefore be interpreted as the result of a trade-off between an increased number of cavitation events and a reduced mechanical intensity per individual collapse.

In the intermediate temperature range (35–55 °C), the system resides in a transition regime between gas-dominated and vapour-dominated cavitation. The concentration of dissolved gases is already significantly reduced, limiting the availability of stable cavitation nuclei, while the vapour pressure is sufficiently high to introduce substantial damping during bubble collapse. At the same time, the reduction in surface tension and viscosity is not yet sufficient to fully compensate for these effects by increasing bubble population. Consequently, both the number of effective cavitation events and the intensity of individual collapses are suboptimal, resulting in a minimum in erosion efficiency.

It should be noted that, despite careful control of experimental conditions, the results exhibit pronounced stochasticity, particularly at elevated temperatures. This is reflected in increased data scatter and is inherent to the probabilistic nature of cavitation, which is governed by nucleation processes and is highly sensitive to microscopic heterogeneities, such as impurities, pre-existing microbubbles, and surface defects. Similar non-monotonic temperature dependences and stochastic behaviour of aluminium cavitation erosion have been reported previously [57,58].

### 2.7. Effect of Surface Tension on the Cavitation Dose

To investigate the effect of surface tension on cavitation-induced dispersion, experiments were carried out using aqueous solutions of sodium dodecyl sulfate (SDS) at various concentrations in an Ultrasonic cleaner operating at 35 kHz with a nominal power density of 60 W·L^−1^ at 25 °C for 5 min (Table 5). As the surface tension of the liquid decreased, the aluminum foil mass loss decreased systematically from 16.4 ± 3.2% to 6.1 ± 2.5%.

Although surfactants are often introduced to facilitate cavitation inception by lowering surface tension, the present results demonstrate that a reduction in surface tension can adversely affect cavitation-induced dispersion efficiency. This behavior can be rationalized in terms of bubble dynamics. Lower surface tension alters the balance between inertial and surface forces acting on oscillating bubbles, which can promote shape instabilities and bubble fragmentation during the expansion stage. As a result, bubbles may split into smaller daughter bubbles before reaching the critical radius required for inertial collapse [24]. Such premature fragmentation limits the energy accumulated during bubble growth and leads to weaker collapse events, thereby reducing the intensity of shock waves and microjets generated upon collapse [21], which ultimately decreases erosion efficiency.

### 2.8. Effect of Solvent Properties on the Cavitation Dose

**Figure 7:**
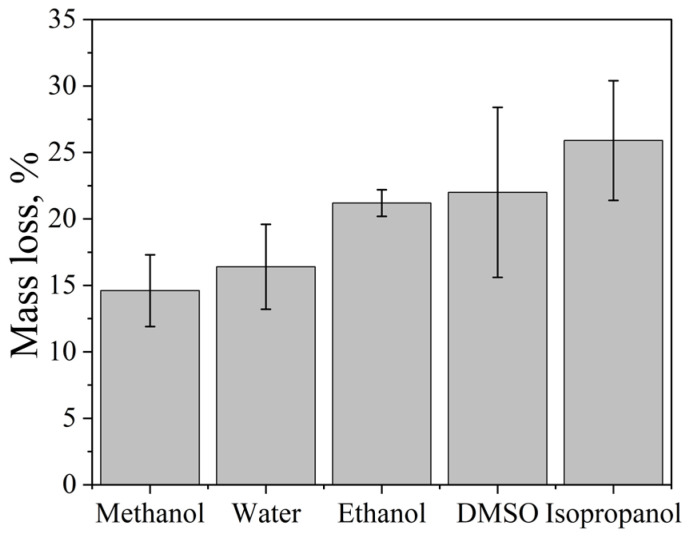
Mass loss of aluminum foil during sonication in various solvents (Ultrasonic cleaner bath, 35 kHz, 60 W·L^−1^, 25 °C, 5 min). Data are presented as mean ± standard deviation (SD), *n* = 12 independent experiments. Error bars represent the SD.

The influence of solvent properties on aluminum foil dispersion was investigated using an Ultrasonic cleaner operating at 35 kHz with a nominal power density of 60 W·L^−1^ at 25 °C for 5 min. The cavitation dose, expressed as aluminum foil mass loss, increased in the following order: methanol < water < ethanol < DMSO < isopropanol (Figure 7). This non-trivial trend reflects the combined influence of several key physicochemical properties of the solvent, including saturated vapor pressure, surface tension, viscosity, gas solubility, and density (Table 6).

Methanol exhibited the lowest dispersion efficiency among the solvents studied. This behavior can be primarily attributed to its relatively high saturated vapor pressure, which favors vapor cavitation. In vapor-filled bubbles, a significant fraction of the collapse energy is dissipated through vapor compression, resulting in a pronounced cushioning effect and reduced mechanical impact on the solid surface [51]. Consequently, the erosion of aluminum foil in methanol is limited.

Water displayed an intermediate cavitation dose. Despite its low vapor pressure, which is favorable for energetic bubble collapse, water possesses the highest surface tension and the lowest gas solubility among the investigated solvents. These properties increase the cavitation onset threshold and limit the number of active cavitation nuclei, leading to a lower density of cavitation events and moderate erosion efficiency.

Ethanol demonstrated a higher mass loss compared to both methanol and water. Its lower saturated vapor pressure relative to methanol suppresses vapor cavitation, while its significantly higher gas solubility facilitates the formation of gaseous cavitation bubbles. These conditions promote more intense inertial collapse and enhanced mechanical erosion of the foil.

DMSO showed a further increase in dispersion efficiency. Its surface tension is approximately half that of water, which reduces the cavitation onset threshold and has been correlated with enhanced sonochemical activity [19]. At the same time, the relatively high viscosity of DMSO slows the motion of the bubble wall during oscillation, extending bubble lifetime and potentially increasing the mechanical impulse delivered upon collapse [26]. The combined effect of reduced cavitation threshold and prolonged bubble dynamics results in a higher cavitation dose compared to water and ethanol.

Isopropanol exhibited the highest aluminum foil mass loss among all tested solvents. Its viscosity is comparable to that of DMSO, while its gas solubility is approximately four times higher. The elevated concentration of dissolved gases provides abundant cavitation nuclei during the rarefaction phase of the acoustic cycle, substantially lowering the cavitation onset threshold. In combination with a moderate vapor pressure that limits vapor cushioning, these factors lead to the most intense mechanical cavitation and maximum erosion efficiency.

**Figure 8:**
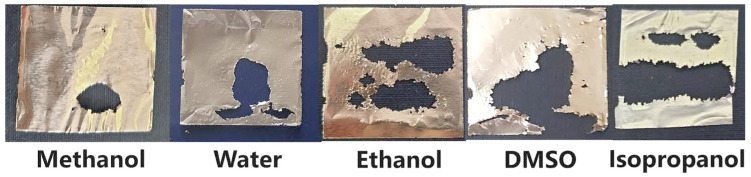
Spatial distribution of aluminum foil erosion patterns after sonication in different solvents, showing localized damage at pressure antinodes.

Additional insight into the cavitation mechanism is provided by the spatial distribution of foil damage within the acoustic field (Figure 8). In several experiments, particularly for ethanol and isopropanol, erosion was strongly localized in regions corresponding to pressure antinodes of the standing ultrasonic wave [15,40]. Such localization is characteristic of mechanically driven cavitation erosion and confirms that shock waves and microjets generated during inertial bubble collapse dominate the dispersion process in these solvents.

Based on both cavitation efficiency and practical considerations, isopropanol emerges as the most suitable solvent among those investigated for cavitation-assisted dispersion of aluminum foil.

## 3. Materials and Methods

### 3.1. Chemicals

Aluminum foil (thickness 10 μm, GOST 745-2014, RUSAL-Sayanal, Sayanogorsk, Russia), sodium dodecyl sulfate (CH_3_(CH_2_)_11_OSO_3_Na, ≥96%, JSC Lenreactiv, Saint Petersburg, Russia), dimethyl sulfoxide ((CH_3_)_2_SO, ≥99.7%, Acros Organics, Fair Lawn, NJ, USA), methanol (CH_3_OH, ≥99.8%, KhimMedService, Moscow, Russia), ethanol (C_2_H_5_OH, 95%, Konstanta-farm M, Odintsovo, Russia), isopropanol ((CH_3_)_2_CHOH, ≥99.7%, Promkhim, Tolyatti, Russia). All chemicals were used as received without further purification.

### 3.2. Cavitation Mapping of Ultrasonic Baths

**Figure 9:**
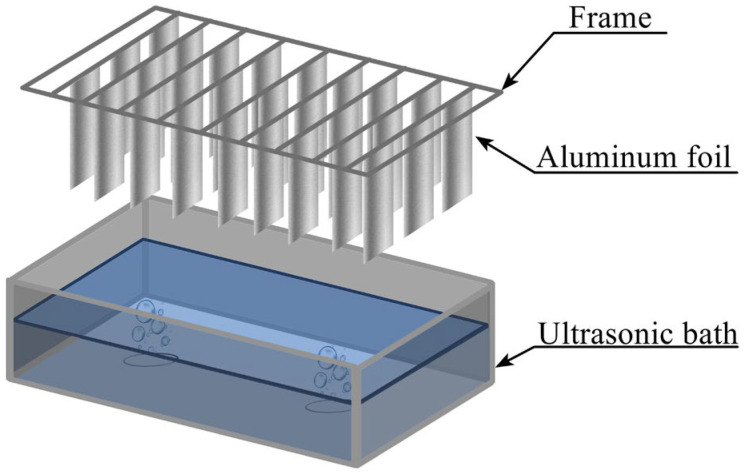
Schematic representation of cavitation mapping setup using aluminum foil in an ultrasonic bath.

Pre-weighed aluminum foil sheets (12 × 2.5 cm) were clamped at the top edge to a wooden frame and positioned vertically in the bath, with the lower edge 10 mm above the bottom and the upper edge at the liquid surface (Figure 9). Ultrasonic treatment was performed for 5 min at a power density of 60 W·L^−1^ in the ultrasonic bath (Ultrasonic cleaner (Shenzhen Chaojie Technology Industrial Co., Ltd., Guandong, China; 35 kHz; single 45 mm transducer) or Vilitek VBS-13DS, Moscow, Russia) (68 kHz; two 45 mm transducers)). After treatment, the foil sheets were removed, rinsed with distilled water, dried at 50 °C in a drying oven, and weighed. Seven replicate runs were performed for each position. Statistical processing of the results was carried out using Statgraphics 18 software. Results are presented as mean ± standard deviation (SD), *n* = 7 independent experiments.

### 3.3. Sonochemical Dispersion of Aluminum Foil in an Ultrasonic Bath

**Figure 10:**
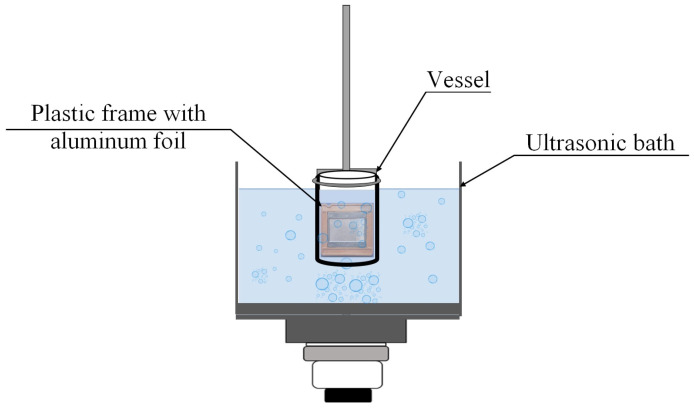
Schematic representation of the setup for sonochemical dispersion of aluminum foil in an ultrasonic bath.

Pre-weighed aluminum foil sheets (3.6 × 3.3 cm) were clamped in a plastic frame and immersed in a 150 mL glass or plastic vessel containing 100 mL of liquid (distilled water, methanol, ethanol, isopropanol, or DMSO). The vessel was placed in the ultrasonic bath (Ultrasonic cleaner or Vilitek VBS-13DS) with the plane of the foil oriented vertically, parallel to the direction of wave propagation and perpendicular to the pressure node and antinode planes (Figure 10). Ultrasonic treatment was performed under various conditions: power density (20–60 W·L^−1^), frequency (35 or 68 kHz), treatment time (3–45 min), and temperature (15–75 °C), as specified in the corresponding figure captions and tables. A summary table (Table S1) detailing all fixed parameters for each experimental series is provided in the Supplementary Information. Temperature was maintained using the bath’s built-in heater or external cooling as required. After treatment, the foil was removed, rinsed with distilled water, dried at 50 °C, and weighed. Twelve replicate runs were performed for each set of parameters. Statistical processing of the results was carried out using Statgraphics 18 software. Results are presented as mean ± standard deviation (SD), *n* = 12 independent experiments.

## 4. Conclusions

In this work, the key parameters of ultrasonic treatment governing the cavitation dose in ultrasonic baths were investigated using aluminum foil mass loss as a quantitative and reproducible metric. All parametric studies were performed at a fixed sample position corresponding to the region of maximum cavitation intensity, ensuring consistent and comparable exposure conditions.

The results demonstrate that dispersion efficiency is governed by a complex interplay between acoustic conditions and the physicochemical properties of the liquid medium, rather than by any single parameter. In addition to ultrasonic frequency and power, the material of the vessel was found to significantly influence the cavitation dose, presumably through its effect on acoustic boundary conditions and energy dissipation at the liquid–solid interface.

A pronounced non-monotonic dependence of cavitation dose on dissolved gas concentration was observed. While moderate gas content promotes cavitation by providing nucleation sites, high gas concentrations (>50 mmol·L^−1^) significantly reduce foil erosion due to the formation of dense cavitation clouds that attenuate ultrasonic wave propagation. Importantly, the investigated parameters are strongly interdependent: increased power and prolonged sonication raise the solvent temperature, which in turn reduces gas solubility and alters cavitation dynamics. This interdependence provides a plausible physical explanation for the stochastic behavior frequently observed in ultrasonic bath experiments.

Contrary to common assumptions, in the present system the addition of surfactants reduced dispersion efficiency, as decreased surface tension disrupts bubble growth and inertial collapse. A complex, non-monotonic temperature dependence was established, with the maximum cavitation dose observed at 15 °C and 65 °C. Among the solvents investigated under identical acoustic and geometrical conditions, isopropanol exhibited the highest dispersion efficiency, which can be attributed to its favorable combination of moderate viscosity, high gas solubility, and relatively low saturated vapor pressure, promoting intense mechanical cavitation.

Overall, this study provides a systematic and quantitative assessment of how key experimental parameters—including acoustic conditions, solvent properties, and vessel material—affect the cavitation dose delivered to a sample in an ultrasonic bath. The results contribute to understanding these complex relationships and offer practical guidelines for improving reproducibility and optimizing cavitation-assisted dispersion processes.

## Figures and Tables

**Table 1:** Characteristics of vessels * [48,49].

Vessel	Material Composition, %	d, mm	E, GPa	β, dB/cm	ν, m/s
1	72 SiO_2_; 5 Al_2_O_3_; 11 Na_2_O; 12 B_2_O_3_	1	48–83	0.01–0.1	5000
2	73 SiO_2_; 2 Al_2_O_3_; 15 Na_2_O; 10 CaO	3			
3	(C_3_H_6_)_n_	1	1.2–1.6	0.5–3.0	1400–1600

* d—wall thickness; E—Young’s modulus; β—attenuation coefficient; ν—speed of sound.

**Table 2:** Dependence of aluminum foil mass loss on ultrasonic frequency.

Ultrasonic Bath	Frequency, kHz	Mass Loss, %
Vilitek VBS-13DS	68	6.4 ± 4.6
Ultrasonic cleaner	35	16.4 ± 1.7

**Table 3:** Dependence of aluminum foil mass loss on dissolved gas concentration [54,55].

№	C_gases_ *, mmol/L	Mass Loss, %
1	<0.01	12.9 ± 3.2
2	0.7	16.4 ± 3.2
3	50	4.1 ± 1.3

* C_gases_—total concentration of dissolved gases (CO_2_; O_2_; N_2_).

**Table 4:** Physical properties * of water at various temperatures.

T, °C	*p,* kg/m^3^	P, kPa	σ, mN/m	η, mPa∙s
15	999.1	1.7	73.5	1.140
25	997.1	3.2	72.0	0.894
35	994.1	5.6	70.4	0.723
45	990.3	9.6	68.7	0.600
55	985.7	15.8	67.1	0.506
65	981.3	25.0	65.4	0.435
75	975.2	38.9	63.6	0.379

* T—temperature; *p*—density; P—vapor pressure; σ—surface tension; η—dynamic viscosity.

**Table 5:** Effect of sodium dodecyl sulfate solution properties * on aluminum foil mass loss.

C, mol/L	σ, mN/m	η, mPa∙s	Mass Loss %
1 × 10^−2^	27	0.894	6.1 ± 2.5
1 × 10^−3^	37	0.894	10.7 ± 2.6
1 × 10^−4^	49	0.894	14.2 ± 3.5
0	74	0.894	16.4 ± 3.2

* C—concentration SDS; σ—surface tension; η—dynamic viscosity.

**Table 6:** Physical properties of solvents * (20 °C) [59,60,61].

Properties of Solvents	Methanol	H_2_O	Ethanol	DMSO	Isopropanol
T_b.p._ °C	65	100	78	189	82
Cp, J/(g∙K)	2.47	4.18	2.43	1.86	2.59
η, mPa∙s	0.8	1.0	1.2	2.5	2.4
σ, mN/m	22.5	72.8	22.4	43.0	21.7
M, g/mol	32	18	46	78	60
*p*, g/cm^3^	0.79	1.0	0.79	1.10	0.78
C_gas_, mM: O_2_/N_2_	11.1/6.1	1.2/0.7	10.9/6.7	2.5/1.2	10.3/5.9
P, kPa	16.90	3.17	7.87	0.06	6.02

* T_b.p._—boiling point solvent; Cp—heat capacity; η—dynamic viscosity; σ—surface tension; M—molar mass; *p*—density solvent; C_gas_—concentration of dissolved gas; P—vapor pressure.

## Data Availability

The original contributions presented in this study are included in the article/Supplementary Material. Further inquiries can be directed to the corresponding author(s).

## Supplementary Materials

The following supporting information can be downloaded at: https://www.mdpi.com/article/10.3390/molecules31081291/s1, Table S1: Summary table of experimental parameters performed in the article; Figure S1: Aluminum foil mass loss as a function of water temperature in ultrasonic-bath (a) Ultrasonic cleaner; (b) Vilitek VBS-13DS bath. Data are presented as mean ± standard deviation (SD), *n* = 12 independent experiments. Error bars represent the SD.

## Abbreviations

The following abbreviations are used in this manuscript:

DMSO	Dimethyl sulfoxide
SDS	sodium dodecyl sulfate
