# Supplementary material for: Effect of Experimental Parameters on Cavitation Dose in Ultrasonic Baths via Modified Aluminum Foil Test
Source: Molecules. 2026 Apr 15;31(8):1291. doi: 10.3390/molecules31081291 (PMC13118934; doi:10.3390/molecules31081291)
Supplement: Supplementary file 1 [file molecules-31-01291-s001.zip › molecules-4193582-supplementary.pdf]

Supplementary Materials

Effect of Experimental Parameters on Cavitation Dose in Ultrasonic Baths via Modified Aluminum Foil Test

Svetlana Saikova [1,2,*], Diana Nemkova [1,2], Anton Krolikov [2]

[1] School of Non-Ferrous Metals, Siberian Federal University, Svobodny, 79, 660041 Krasnoyarsk, Russia;
dsaykova@sfu-kras.ru (D.N.); antonkrolikov@mail.ru (A.K.);

[2] Institute of Chemistry and Chemical Technology, Federal Research Center "Krasnoyarsk Science Center of the Siberian Branch of the Russian Academy of Sciences", Akademgorodok, 50/24, 660036 Krasnoyarsk, Russia; ssai@mail.ru (S.S.)

* Correspondence: ssaikova@sfu-kras.ru

Supplementary Materials: The following supporting information can be downloaded at: www.mdpi.com/xxx/s1, Table S1: Summary table of experimental parameters performed in the article; Figure S1: Aluminum foil mass loss as a function of water temperature in ultrasonic-bath (a) Ultrasonic cleaner; (b) Vilitek VBS-13DS bath, data are presented as mean ± standard deviation (SD), n = 12 independent experiments. Error bars represent the SD.

Author Contributions: Conceptualization, D.N. and S.S.; methodology, S.S.; software, A.K.; validation, S.S., D.N.; formal analysis, D.N., S.S. A.K; investigation, D.N.; writing—original draft preparation, S.S., D.N. A.K; writing—review and editing, S.S.; visualization, A.K.; supervision, S.S.; project administration, S.S. All authors have read and agreed to the published version of the manuscript.

Funding: This research was funded by the Government Assignment to the Institute of Chemistry and Chemical Engineering, Siberian Branch of the Russian Academy of Sciences (project No. FWES-2026-0004).

Data Availability Statement: Not applicable

Acknowledgments: This research was carried out using the equipment of Krasnoyarsk Regional Center of Research Equipment of Federal Research Center "Krasnoyarsk Science Center SB RAS" and Center for Collective Use "Science-intensive methods of research and analysis of new materials, nano-materials and mineral raw materials" of Siberian Federal University.

Conflicts of Interest: The authors declare no conflict of interest. The funders had no role in the design of the study; in the collection, analyses, or interpretation of data; in the writing of the manuscript; or in the decision to publish the results.

Table S1. Summary table of experimental parameters performed in the article

	Ultrasonic power density W/L	Treatment time, min	Solvent:	Temperature, °C	Dissolved gas concentration:	Vessel	Frequenc y, kHz	C_{SDS}, mM
**Power effect**	**60, 54, 48, 42, 36, 30, 24, 18**	5	distilled water	25 ± 1	0.7	glass tank (150, 1 mm wall)	35	0
**Time effect**	60	**0, 3, 5, 15, 25, 30, 35, 45**	distilled water	25 ± 1	0.7	glass tank (150, 1 mm wall)	35	0
**Solvent effect**	60	5	**distilled water, methanol, ethanol, isopropanol, DMSO**	25 ± 1	0.7	glass tank (150, 1 mm wall)	35	0
**Temperature effect**	60	5	distilled water	**15, 25, 35, 45, 55, 65, 75 °C**	0.7 (no re-equilibrated at each temperature)	glass tank (150, 1 mm wall)	35	0
**Dissolved gas concentration effect**	60	5	distilled water	60	**<0.01 0.7 50**	glass tank (150, 1 mm wall)	35	0
**Dissolved gas concentration effect**	60	5	distilled water	25 ± 1	0.7	**glass tank (150, 1 mm wall), glass tank (150, 3 mm wall), plastic tank (150, 1 mm wall),**	35	0
**Frequency effect**	60	5	distilled water	25 ± 1	0.7	glass tank (150 ml, 1 mm wall)	**35, 68**	0
**Surface tension effect**	60	5	distilled water	25 ± 1	0.7	glass tank (150, 1 mm wall)	35	**10, 1, 0.1**

* C_{gases} - total concentration of dissolved gases (CO_2; O_2; N_2).

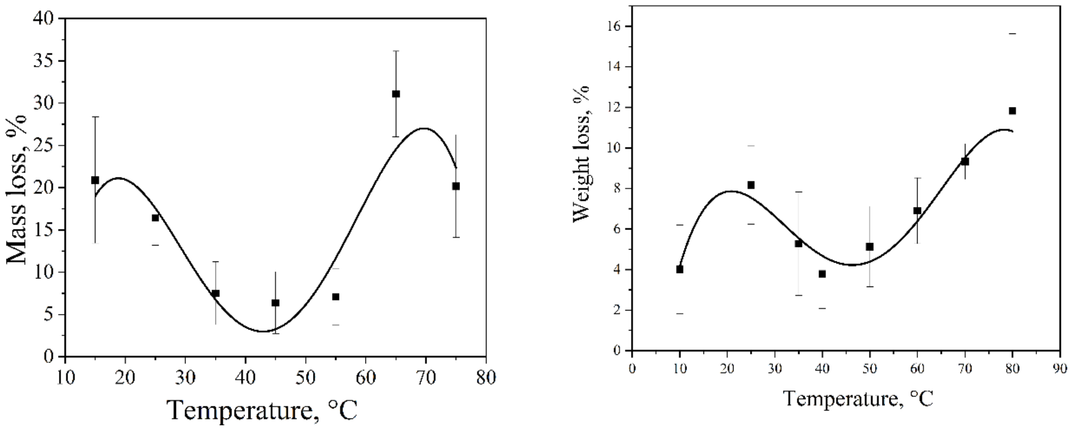

(a) (b)

Figure S1. Aluminum foil mass loss as a function of water temperature in ultrasonic-bath (a) Ultrasonic cleaner; (b) Vilitek VBS-13DS bath, data are presented as mean ± standard deviation (SD), n = 12 independent experiments. Error bars represent the SD.
